# Leptin in Dental Pulp and Periapical Tissues: A Narrative Review

**DOI:** 10.3390/ijms23041984

**Published:** 2022-02-11

**Authors:** Jenifer Martin-Gonzalez, Juan J. Segura-Egea, Antonio Pérez-Pérez, Daniel Cabanillas-Balsera, Víctor Sánchez-Margalet

**Affiliations:** 1 Endodontic Section, Department of Stomatology, University of Sevilla, C/Avicena s/n, 41009 Sevilla, Spain; segurajj@us.es; 2 Department of Clinical Biochemistry, Virgen Macarena University Hospital, University of Sevilla, 41009 Sevilla, Spain; aperez14@us.es (A.P.-P.); margalet@us.es (V.S.-M.)

**Keywords:** leptin, leptin receptor, dental pulp, periapical tissues, DMP-1, DSPP, vital pulp therapy, regenerative endodontic procedures

## Abstract

Leptin is a non-glycosylated 16 kDa protein synthesized mainly in adipose cells. The main function of leptin is to regulate energy homeostasis and weight control in a central manner. There is increasing evidence that leptin also has systemic effects, acting as a link between innate and acquired immune responses. The expression of leptin and its receptor in human dental pulp and periradicular tissues have already been described, as well as several stimulatory effects of leptin protein expression in dental and periodontal tissues. The aim of this paper was to review and to compile the reported scientific literature on the role and effects of leptin in the dental pulp and periapical tissues. Twelve articles accomplished the inclusion criteria, and a comprehensive narrative review was carried out. Review of the available scientific literature concluded that leptin has the following effects on pulpal and periapical physiology: 1) Stimulates odontogenic differentiation of dental pulp stem cells (DPSCs), 2) Increases the expression of dentin sialophosphoprotein (DSPP) and dentin matrix protein-1 (DMP-1), odontoblastic proteins involved in odontoblastic differentiation and dentin mineralization, 3) Stimulates vascular endothelial growth factor (VEGF) expression in human dental pulp tissue and primary cultured cells of human dental pulp (hDPCs), 4) Stimulates angiogenesis in rat dental pulp cells, and 5) Induces the expression of interleucinas 6 and 8 in human periodontal ligament cells (hPDLCs). There is evidence which suggests that leptin is implicated in the dentin mineralization process and in pulpal and periapical inflammatory and reparative responses.

## 1. Introduction

At present, the possible association between oral inflammatory processes caused by infection and systemic health is an interesting aspect that the medical and dental community focuses its research on to improve the health of patients [1]. In spite of several epidemiological studies that have shown this association [2,3,4,5,6,7,8], the basic, molecular and cellular aspects of the pathophysiology of oral inflammatory processes caused by infection have not been fully researched. 

Pulpitis and apical periodontitis are oral inflammatory processes caused by infection, and these diseases may help the scientific community as they are ideal models to explore the pathways or mechanisms by which oral local infections can harm systemic health. 

The dental pulp is a connective tissue that is located inside of the tooth and is limited by dentin, a hard, calcified and continuous formation tissue. The dental pulp and dentin are two tissues with different histological characteristics, but due to their similar embryological origin and structural implications are considered a functional unit. To establish a correct knowledge of dental pulp biology, it is necessary to also know the tissues that surround it; both dentin and periapical tissues are intimately involved in pathophysiology of the dental pulp [9]. When etiological agents invade the dental pulp an immune and inflammatory response is stimulated and pulpitis or dental pulp inflammation appears [10]. Pulpitis is understood as a dynamic process in which etiological agents invade (these are the main cause of pulpy injury and the bacteria that cause tooth caries) and the immune and inflammatory host responds [11]. If the intensity of the aggressive factors is mild or moderate, a reversible pulpitis will be established and pulp inflammation will be controlled and repaired, maintaining pulp sensitivity. If the intensity of the aggressive factors is severe, an irreversible pulpitis will be induced and, ultimately, pulp necrosis and damage to the dental pulp will be not repaired [9]. 

The inflammation of the tissue around the tooth root, usually around the root apex, caused by infection of the dental pulp, is known as apical periodontitis [12]. The prevalence of apical periodontitis is very high in the worldwide population [13]. In most cases, apical periodontitis occurs when cariogenic microorganisms or antigenic content that have invaded the dental pulp reach the periapical or periradicular connective tissue, stimulating an inflammatory and immune response. As pulpitis, the apical periodontitis can be reversible or irreversible and an acute or chronic inflammation. Acute and symptomatic apical periodontitis is characterized by minimal pain and bone resorption and is sometimes reversible. Chronic apical periodontitis is always irreversible, as a consequence of necrotic pulp, and is characterized by bone destruction with radiologically observable periapical or peri-radicular osteolytic lesions [9].

The link between pulp/apical inflammation and pulp/apical repair is still not fully understood. The prerequisite for tissue repair is the host’s inflammatory response, which includes the production of antimicrobial peptides and cytokines and the activation of migratory immune cells [14]. Leptin, as a pro-inflammatory cytokine, could influence periapical and pulp defensive and reparative responses.

The aim of this review is to analyze the available scientific evidence about the expression and effects of leptin in dental pulp and periapical tissues in order to clarify and summarize the role of leptin in pulpal and periapical physiology.

## 2. Literature Search and Scope of The Review

An electronic search of PubMed, Web of Science, and Scopus was conducted using appropriate keywords, as follows: (leptin OR leptin receptor OR LEPR OR OBR OR OB-R OR Ob-Rb) AND (dental pulp OR periapical tissues OR apical granuloma OR apical abscess OR periapical abscess OR periapical granuloma OR apical cyst OR periapical cyst OR periapical granuloma OR periradicular tissues OR pulpitis OR apical periodontitis). All studies which carried out investigations of the expression of leptin or LEPR, as well as leptin effects, in dental pulp tissue or in periapical tissues were included. Fifteen articles were applicable to these inclusion criteria. Taking into account the scope of this search and the type of studies found, a comprehensive narrative review was carried out.

## 3. Leptin

Leptin, whose first functional role is the control of appetite and hunger [15], is a non-glycosylated hormone composed of 146 amino acids [16] synthesized mainly in adipose cells [17]. The amount of energy stored in the adipose tissue and the body adipose mass is proportional to the amount of circulating leptin (normal range 1–15 ng/mL). People with obesity produce more leptin than those of a normal weight [18]. Increasing amounts of evidence have shown that leptin has systemic effects, apart from those related to energy homeostasis, including the regulation of neuroendocrine and reproductive, hematopoietic and immune functions [19,20,21]. The discovery of leptin as a proinflammatory adipokine forged the new era of immunometabolism [15]. During acute infection and inflammation, leptin levels increase regulating both innate and adaptive immune responses [22,23]. In this sense, leptin is involved in inflammatory/immune-related processes, e.g., by stimulating the proliferation of circulating monocytes [24]. In polymorphonuclear cells, leptin inhibits apoptosis [24,25], promoting chemotaxis [26,27], and improves the expression of CD11b via monocytes by releasing TNF-α [28], as well as stimulating the production of reactive oxygen species (ROS) [29]. Moreover, although the mechanisms by which leptin regulates the T cell function are not fully understood, it has been reported that leptin actions in T cell populations involve different processes, leading to an increase in immune activity by enhancing the polarization of naive T helper cells to a Th1 phenotype. Additionally, leptin leads dendritic cell (DC) differentiation and survival [30] and helps to improve both the activation and proliferation of CD4+ and CD8+ T cells [31,32], as well as promoting Th17 differentiation [33,34] and Th2 responses [35]. By contrast, this hormone reduces the levels of regulatory T cells (Tregs) [36,37] and induces immunosenescence in B cells, decreasing the production of antibodies [38,39].

Leptin acts as a link between obesity and inflammation [40]. The link between leptin and dental pulp/periapical defensive and reparative responses could provide new evidence of the relationship between obesity, inflammation and oral infections. Scientific evidence is emerging that implicates leptin in oral biology and, specifically, in pulpal and periapical physiology. It has been reported that leptin and leptin receptor (LEPR) are expressed in healthy and inflamed gingival tissues [41], and elevated serum leptin concentration has been associated with increased chronic periodontitis [42]. Additionally, it has been shown that rats with high-fructose diets display increased intestinal leptin levels 28 days after apical periodontitis induction [43]. Leptin is implicated in the regeneration and repair of dental structures [44] through the differentiation of dental stem cells from periodontal ligament (PDL) and dental pulp into odontoblast-like cells, preventing their differentiation into adipocytes [45,46]. Moreover, compelling evidence has implicated leptin in dental inflammatory and immune responses [40,46]. 

## 4. Leptin and The Dental Pulp

El Karim et al. (2009) [47] were the first to report that leptin is synthesized and secreted in vitro by pulp fibroblasts derived from extracted healthy molar teeth (Table 1). Posteriorly, it was reported that leptin is expressed by SCs derived from human natal dental pulp (hNDP) [48], ameloblasts, odontoblasts, dental papilla cells and stratum intermedium cells in rat and human tooth germs at the late bell stage, in rat dental pulp [49], in the dental pulp of monkeys and human primary cultured cells [50]. However, other dental pulp cells might also be a source of leptin. In this sense, although adipocytes are not a normal cellular component in dental pulp, human dental pulp stem cells (DPSCs) are capable of differentiating into oil red-O-positive lipid-containing adipocytes [51], expressing in vitro the adipogenic master genes *peroxisome proliferator- activated receptor gamma two (PPARγ2)* and *lipoprotein lipase* (*LPL*), two adipocyte-specific transcripts [52]. Therefore, pulpal leptin could be secreted by DPSCs suffering adipogenic differentiation. 

Proposing that leptin has biological functions in the dental pulp requires demonstrating that LEPR, the specific receptor for leptin, is expressed in pulpal cells. The expression of LEPR in human dental pulp was first described by Martín-González et al. [53] (Table 1). The odontoblast layer of human dental pulp was immunoreactive for LEPR [54], suggesting that human odontoblasts express LEPR. Both leptin and LEPR are expressed by healthy and inflamed human dental pulp cells [53,55], being widely distributed in the dental pulp of human and monkey primates [56]. Moreover, there is an upregulation of leptin and LEPR expression in inflamed human dental pulp [40,53,55]. All these findings suggest that leptin, by autocrine or paracrine pathways, could play a role in dental pulp physiology.

The effects of leptin on dental pulp have been investigated. The results of the studies carried out suggest that leptin is involved in the odontogenic differentiation of DPSCs, dentin matrix mineralization, and angiogenesis (Table 2).

Leptin acts as a modulator of pulpal mesenchymal stem cell differentiation, promoting odontoblastic and suppressing adipogenic differentiation [57,58]. On the other hand, leptin stimulates, in a dose-dependent manner, the expression of dentin sialophosphoprotein (DSPP) in human dental pulp [59,60,61]. DSPP acts as nucleator of apatite crystal formation in the presence of collagen during dentin mineralization [62], inducing highly organized intrafibrillar collagen mineralization [63]. Taking into account that DSPP is a marker of odontoblastic differentiation [64,65], this data further supports the concept that human odontoblasts express LEPR [66]. 

Leptin also stimulates the expression of dentin matrix protein-1 (DMP-1) in human dental pulp [60,61]. DMP-1 is an extracellular matrix protein, released by odontoblasts, that stimulates the deposition of mineral particles along the collagen fibril axis [63]. The mitogen-activated protein kinase (MAPK) signaling pathway is involved in leptin-mediated DMP-1 expression in hDPCs [60,61]. On the contrary, the stimulatory effect of leptin on DSPP expression seems to be mediated by both the MAPK [61] and Phosphatidylinositol-3-kinase (PI3K) [60] pathways.

The stimulatory effect of leptin on DSPP and DMP-1 expression in human dental pulp, two critical proteins for proper mineralization of dentin and odontoblast differentiation [67], suggests a functional role for leptin in dentinogenesis and dental pulp regenerative and reparative processes. The especially strong immunoreaction for leptin and LEPR in junctional epithelium, the front-line defense around teeth, and in mineralizing areas of the dental pulp [50], further support the role of leptin in the pulpal defensive response against caries. Additionally, leptin induces mineralization, not only osteodentin but also tubular dentin, in rat pulp cavity after pulp capping [44]. On the other hand, leptin may upregulate interleucinas 6 and 8 (IL-6 and IL-8) production through binding with LEPR in human dental pulp fibroblasts via the activation of different intracellular signaling pathways [68].

**Figure 1:**
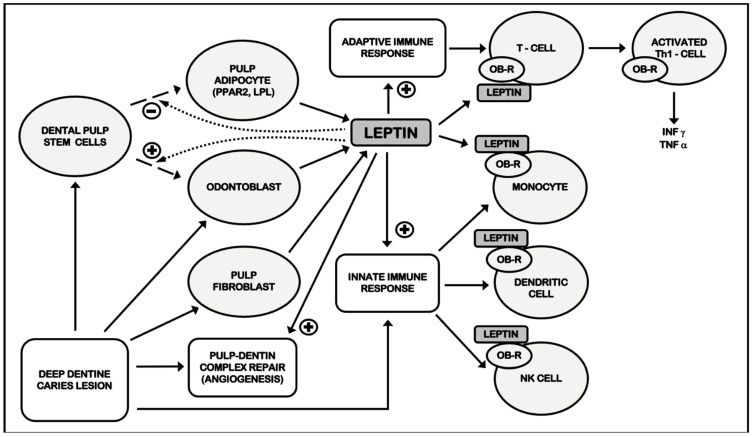
**Summary scheme. Role of leptin in pulpal and periapical pathophysiology.** Leptin levels increase in inflamed human dental pulp (Martín-González et al., 2013a). Leptin is produced by dental pulp fibroblasts (El Karim et al., 2009), odontoblasts (Ide et al., 2011), SCs from human natal dental pulp (Karaöz et al., 2010), and DPSCs suffering adipogenic differentiation (Gronthos et al., 2002). Leptin inhibits DPSCs adipose differentiation and stimulates DPSCs odontoblastic differentiation (dashed arrows) (Um et al., 2011; Choi et al., 2019). Leptin increases DSPP (Martín-González et al., 2015a, Ngo et al., 2018), DMP-1 (Ngo et al., 2018; Martín-González et al., 2019) and VEGF expression (Ngo et al., 2018), promoting matrix mineralization (dentinogenesis), odontoblastic differentiation and angiogenesis. Finally, leptin stimulates the expression of other cytokines (Ngo et al., 2018; Wei et al., 2019) through binding with its receptor (Wei et al., 2019). As a result, leptin could contribute to pulpal and periapical inflammation and repair. DPSCs: dental pulp stem cells. DSPP: dentin sialophosphoprotein. DMP-1: dentin matrix protein-1. SCs: stem cells. VEGF: vascular endothelial growth factor. IL-6: interleukin 6. IL-8: interleukin 8.

Another effect of leptin on pulp physiology is angiogenesis stimulation [58] by increasing vascular endothelial growth factor (VEGF) expression in hDPCs, which is also mediated by the MAPK signaling pathway [61]. Angiogenic growth factors, such as VEGF, are present in human dental pulp and dentin matrix, being upregulated in dental pulp from carious teeth [69]. Angiogenesis is critical and a prerequisite for successful pulpal repair after injury and inflammation, playing a main role in the replacement of damaged tissues during regenerative endodontic procedures (REPs) [70]. Angiogenesis also promotes root development and apical root closure in cases of immature teeth [71]. As a result, by regulating the expression of VEGF, leptin could play an important role in angiogenesis during pulpitis and REPs, similar to that which has already been described in other inflamed tissues and cancer [72]. Summary scheme addressing the pathophysiological role of leptin in dental and periapical pulp is shown in the following (Figure 1):

## 5. Leptin and Periradicular Tissues

The first studies that linked leptin with periradicular tissues focused on their possible relationship with periodontal disease. Johnson and Serio [41] described the expression of leptin and LEPR in healthy and inflamed gingival tissues. More recently, a high serum concentration of leptin has been associated with generalized chronic periodontitis [42], with leptin being considered as a possible marker of inflammatory activity in chronic periodontitis [43]. 

Regarding the presence of leptin and its receptor in periapical tissues, the expression of both leptin and LEPR in human chronic periapical lesions was first described by Kangarlou et al. [73] (Table 1). Later, the presence of leptin [53] and LEPR [55] in human chronic granulomatous inflammatory tissues was demonstrated by immunohistochemistry. Amongst the inflammatory cells present in the periapical granulomas, only macrophages were reactive to leptin antibodies [54]. The presence of leptin and LEPR mRNAs, determined by a quantitative real-time PCR (qRT-PCR), and leptin and LEPR proteins, analyzed by immunoblot, have also been demonstrated in human periapical granulomas [54,60].

About the possible role of leptin in periradicular tissues, few data are available. Leptin and LEPR increase substantially in inflammatory periradicular tissues, inducing the expression of mRNA and the proteins IL-6 and IL-8 in human periodontal ligament cells (hPDLCs); these effects correlate with the extent of inflammatory infiltration [56] (Table 2). On the contrary, leptin and LEPR are downregulated by inflammatory signals, interfering negatively with the regenerative capacity of human PDL cells [74]. These results, taken together with those previously described in relation to dental pulp, suggest that leptin could play a role in inflammatory and immune periapical responses.

This is comparable to what happens in obesity [75], where inflammatory cells such as neutrophils, eosinophils, and macrophages are abundant in inflamed pulpal and periapical tissue [76]. Although bacterial antigens, the lipopolysaccharide of gram-negative bacteria (LPS) and lipoteichoic acid of gram-positive bacteria (LTA), are the primary cause of both pulpal and periapical inflammation, the immune response of the host determines the development of the inflammatory response in dental pulp and periapical tissue. Lymphocyte trafficking into pulpal and periapical inflamed tissues is essential in these responses, and is regulated by chemokines such as CC-chemokine ligand 20 (CCL20) and the recruitment of memory T cells [77] and immature dendritic cells [78]. Taking into account the upregulation of leptin in inflamed tissues, such as dental pulp [59], the high levels of this adipokine demonstrated in chronic periapical lesions [55] and the potent stimulatory effect of leptin on CCL20 expression [78], it could be suggested that leptin modulates the production of chemotactic signals and the trafficking of lymphocytes during pulpal and periapical inflammatory processes.

## 6. Biological Significance of The Relationship between Leptin and Pulpal-Periapical Physiology

The functional effects mediated by the interaction of leptin with its specific receptor LEPR in dental pulp and periradicular tissues, described in this review, suggest that leptin is involved in the biomineralization process and in pulpal and periapical inflammatory and reparative responses. However, the biological significance of the link between pulpal and periapical physiology and an adipokine, such as leptin, mainly released by adipose tissue and whose first functional role is the control of appetite and hunger [15,16], need to be analyzed and clarified. 

The fact that leptin and LEPR are expressed in pulpal and periapical tissues could be interpreted as evidence supporting the relationship between obesity, inflammation, and endodontic infections. Several possible pathways involving the host response and bacterial challenge have been proposed to explain the association between obesity and inflammation [79]. Obesity could alter the host immune response to oral infection, increasing individuals’ susceptibility [80]. Inflammatory cells (T-lymphocytes, macrophages, neutrophils) and pro-inflammatory cytokines (IL-6, IL-8) which are implicated in the pathogenesis of endodontic infections, such as pulpitis and apical periodontitis, could be influenced by obesity and leptin levels [80,81]. The altered inflammatory state in patients with obesity could predispose individuals to increased periapical tissue destruction, impairing periapical healing after root canal treatment (RCT) [82]. The possible biological mechanisms involved in the worst prognosis of root-filled teeth in diabetic patients, with higher frequency of persistent apical periodontitis after RCT [83] and non-retention of root-filled teeth [84], have been reviewed [85,86]. Taking into account that obesity is significantly more frequent in diabetics [87], obesity, through leptin levels, could be another biological mechanism linking diabetes and the outcome of RCT. 

## 7. Highlights

Several important effects of leptin in pulpal and periapical physiology have been described: (1) Stimulates odontogenic differentiation of DPSCs, (2) Increases the expression of DSPP and DMP-1, odontoblastic proteins involved in dentin mineralization and odontoblastic differentiation, (3) Stimulates VEGF expression in human dental pulp tissue and primary cultured cells of human dental pulp (hDPCs), (4) Stimulates angiogenesis in rat dental pulp cells, and (5) Induces the expression of IL-6 and IL-8 in human periodontal ligament cells (hPDLCs).

## 8. Conclusions

The data provided identify that there is evidence regarding the role of leptin in the dentin mineralization process and/or in pulpal and periapical inflammatory and reparative responses. The information is relevant and potentially contributes to this field of study; however, the little information that exists to date in this line of research may prove a limitation. Further research is needed to fully discern the possible connection between obesity, inflammation and endodontic infections and should be investigated.

## Figures and Tables

**Table 1:** Leptin and LEPR expression in dental pulp and periradicular tissues.

Authors/Year	Leptin/LEPR	Cell/Tissue
El Karim et al., 2009	Leptin	Human dental pulp fibroblasts
Karaöz et al., 2010	Leptin	SCs derived from human natal dental pulp
Kangarlou et al., 2010	Leptin	Human chronic periapical lesions
Ide et al., 2011	Leptin	Ameloblasts, odontoblasts in rat dental pulp
Martín-González et al., 2013a	Leptin	Human dental pulp (healthy and inflamed)
Li et al., 2014	Leptin	Dental pulp of monkeys
		Primary cultured human dental pulp cells
Martín-González et al., 2015b	Leptin	Human periapical granuloma
Kangarlou et al., 2010	LEPR	Human chronic periapical lesions
Martín-González et al., 2013b	LEPR	Human dental pulp (healthy and inflamed)
Li et al., 2014	LEPR	Dental pulp of monkeys
		Primary cultured human dental pulp cells
Martín-González et al., 2015c	LEPR	Human periapical granuloma
Martín-González et al., 2015a	LEPR	Odontoblast layer of human dental pulp

**Table 2:** Leptin effects on pulpal and periapical physiology.

Authors/Year	Tissue	Effect
Um et al., 2011	Pulpal mesenchymal	Promotes odontoblastic differentiation
	stem cells	Suppresses adipogenic differentiation
Li et al., 2015	Human periodontal	Stimulates IL-6 and IL-8 expression ligament cells
Martín-González et al., 2015a	Human dental pulp	Stimulates DSPP expression
Ngo et al., 2018	Human dental pulp	Stimulates DSPP expression
Ngo et al., 2018	Human dental pulp	Stimulates DMP1 expression
Ngo et al., 2018	Human dental pulp	Stimulates VEGF expression
Martín-González et al., 2019	Human dental pulp	Stimulates DSSP expression (PI3K)
		Stimulates DMP-1 expression (MAPK 1/3)
Choi et al., 2019	Rat dental pulp	Stimulates odontoblastic differentiation
		Induces angiogenesis
		Induces mineralization
Wei et al., 2019	Human dental pulp	Upregulates IL-6 and IL-8
	fibroblasts	

DSPP: dentin sialophosphoprotein. DMP-1: dentin matrix protein-1. VEGF: vascular endothelial growth factor. IL-6: interleukin 6. IL-8: interleukin 8. MAPK: mitogen-activated protein kinases pathway. PI3K: phosphatidylinositol 3-kinase pathway.

## Data Availability

The data that support the findings of this study are available from the corresponding author upon.

## References

[1] Seymour R.A. (2009). Is gum disease killing your patient?. Br. Dent. J..

[2] Soskolne W.A. (1998). Epidemiological and clinical aspects of periodontal diseases in diabetics. Ann. Peridontol..

[3] Janket S.J., Baird A.E., Chuang S.K., Jones J.A. (2003). Meta-analysis of periodontal disease and risk of coronary heart disease and stroke. Oral Surg. Oral Med. Oral Pathol. Oral Radiol. Endod..

[4] Jiménez-Pinzón A., Segura-Egea J.J., Poyato M., Velasco E., Ríos J.V. (2004). Prevalence of apical periodontitis and frequency of root-filled teeth in an adult Spanish population. Int. Endod. J..

[5] Segura-Egea J.J., Jiménez-Pinzón A., Ríos-Santos J.V., Velasco-Ortega E., Cisneros-Cabello R., Poyato-Ferrera M. (2005). High prevalence of apical periodontitis amongst type 2 diabetic patients. Int. Endod. J..

[6] Caplan D.J., Chasen J., Krall E.A., Cai J., Kang S., Garcia R.I., Offenbacher F., Beck J.D. (2006). Lesions of endodontic origin and risk of coronary heart disease. J. Dent. Res..

[7] Ridao-Sacie C., Segura-Egea J.J., Fernández-Palacín A., Bullón-Fernández P., Ríos-Santos J.V. (2007). Radiological assessment of periapical status using the periapical index (PAI): Comparison of periapical radiography and digital panoramic radiography. Int. Endod. J..

[8] Segura-Egea J.J., Martín-González J., Cabanillas-Balsera D., Fouad A.F., Velasco-Ortega E., López-López J. (2016). Association between diabetes and the prevalence of radiolucent periapical lesions in root-filled teeth: Systematic review and meta-analysis. Clin. Oral Investig..

[9] Canalda Sahli C., Brau Aguadé E. (2016). Endodoncia: Técnicas Clínicas y Bases Científicas.

[10] Jontell M., Okiji T., Dahlgren U., Bergenholtz G. (1998). Immune defense mechanisms of the dental pulp. Crit. Rev. Oral Biol. Med..

[11] Bergenholtz G. (2000). Evidence for bacterial causation of adverse pulpal responses in resin-based dental restorations. Crit. Rev. Oral Biol. Med..

[12] Eriksen H.M., Ørstavik D., Pitt Ford T.R. (1998). Epidemiology of apical periodontitis. Essential Endodontology: Prevention and Treatment of Apical Periodontitis.

[13] Cabanillas-Balsera D., Segura-Egea J.J., Bermudo-Fuenmayor M., Martín-González J., Sánchez-Jiménez M.C., Areal-Quecuty V., Sánchez-Domínguez B., Montero-Miralles P., Velasco-Ortega E. (2020). Smoking and Radiolucent Periapical Lesions in Root Filled Teeth: Systematic Review and Meta-Analysis. J. Clin. Med..

[14] Horst O.V., Horst J.A., Samudrala R., Dale B.A. (2000). Caries induced cytokine network in the odontoblast layer of human teeth. BMC Immunol..

[15] Flier J.S. (1995). The adipocyte: Storage depot or node on the energy information superhighway?. Cell.

[16] Zhang Y., Proenca R., Maffei M., Barone M., Leopold L., Friedman J.M. (1994). Positional cloning of the mouse obese gene and its human homologue. Nature.

[17] Maffei M., Fei H., Lee G.H., Dani C., Leroy P., Zhang Y., Proenca R., Negrel R., Ailhaud G., Friedman J.M. (1995). Increased expression in adipocytes of ob RNA in mice with lesions of the hypothalamus and with mutations at the db locus. Proc. Natl. Acad. Sci. USA.

[18] Frederich R.C., Hamann A., Anderson S., Löllmann B., Lowell B.B., Flier J.S. (1995). Leptin levels reflect body lipid content in mice: Evidence for diet-induced resistance to leptin action. Nat. Med..

[19] Ahima R.S., Flier J.S. (2000). Leptin. Annu. Rev. Physiol..

[20] Pérez-Pérez A., Sánchez-Jiménez F., Maymó J., Dueñas J.L., Varone C., Sánchez-Margalet V. (2015). Role of leptin in female reproduction. Clin. Chem. Lab. Med..

[21] Pérez-Pérez A., Vilariño-García T., Fernández-Riejos P., Martín-González J., Segura-Egea J.J., Sánchez-Margalet V. (2017). Role of leptin as a link between metabolism and the immune system. Cytokine Growth Factor Rev..

[22] Gualillo O., Eiras S., Lago F., Diéguez C., Casanueva F.F. (2000). Elevated serum leptin concentrations induced by experimental acute inflammation. Life Sci..

[23] Sánchez-Margalet V., Martín-Romero C., Santos-Alvarez J., Goberna R., Najib S., Gonzalez-Yanes C. (2003). Role of leptin as an immunomodulator of blood mononuclear cells: Mechanisms of action. Clin. Exp. Immunol..

[24] Santos-Álvarez J., Goberna R., Sánchez-Margalet V. (1999). Human leptin stimulates proliferation and activation of human circulatingmonocytes. Cell. Immunol..

[25] Bruno A., Conus S., Schmid I., Simon H.-U. (2005). Apoptotic pathways are inhibited by leptin receptor activation in neutrophils. J. Immunol..

[26] Conus S., Bruno A., Simon H.-U. (2005). Leptin is an eosinophil survival factor. J. Allergy Clin. Immunol..

[27] Martín-Romero C., Santos-Álvarez J., Goberna R., Sánchez-Margalet V. (2000). Human leptin enhances activation and proliferation ofhuman circulating T lymphocytes. Cell. Immunol..

[28] Cadelfie-Chezet F., Poulin A., Vasson M.P. (2003). Leptin regulates functional capacities of polymorphonuclear neutrophils. Free Radic. Res..

[29] Cadelfie-Chezet F., Poulin A., Tridon A., Sion B., Vasson M.P. (2001). Leptin: A potential regulator of polymorphonuclear neutrophilbactericidal action?. J. Leukoc. Biol..

[30] Zarkesh-Esfahani H., Pockley G.A., Wu Z., Hellewell P.G., Weetman A.P., Ross R.J.M. (2004). Leptin indirectly activates humanneutrophils via induction of TNF-alpha. J. Immunol..

[31] Suzuki A., Leland P., Joshi B.H., Puri R.K. (2015). Targeting of IL-4 and IL-13 receptors for cancer therapy. Cytokine.

[32] Mattioli B., Straface E., Quaranta M.G., Giordani L., Viora M. (2005). Leptin promotes differentiation and survival of human dendriticcells and licenses them for Th1 priming. J. Immunol..

[33] Kim S.Y., Lim J.H., Choi S.W., Kim M., Kim S.-T., Kim M.-S., Cho Y.S., Chun E., Lee K.-Y. (2010). Preferential effects of leptin on CD4T cells in central and peripheral immune system are critically linked to the expression of leptin receptor. Biochem. Biophys. Res. Commun..

[34] Moon J., Kim D., Kim E.K., Lee S.Y., Na H.S., Kim G.N., Lee A., Jung K., Choi J.W., Park S.H. (2020). Brown adipose tissue ameliorates autoimmune arthritis via inhibition of Th17 cells. Sci. Rep..

[35] Reis B.S., Lee K., Fanok M.H., Mascarague C., Amoury M., Cohn L.B., Rogoz A., Dallner O.S., Moraes-Vieira P.M., Domingos A.I. (2015). Leptin receptor signaling in T cells is required for Th17 differentiation. J. Immunol..

[36] Zheng H., Zhang X., Castillo E.F., Luo Y., Liu M., Yang X.O. (2016). Leptin enhances Th2 and ILC2 responses in allergic airwaydisease. J. Biol. Chem..

[37] De Rosa V., Procaccini C., Calì G., Pirozzi G., Fontana S., Zappacosta S., La Cava A., Matarese G. (2007). A key role of leptin in thecontrol of regulatory T cell proliferation. Immunity.

[38] Matarese G., Procaccini C., De Rosa V., Horvath T.L., La Cava A. (2010). Regulatory T cells in obesity: The leptin connection. Trends Mol. Med..

[39] Frasca D., Díaz A., Romero M., Blomberg B.B. (2020). Leptin induces immunosenescence in human B cells. Cell. Immunol..

[40] Fernández-Riejos P., Najib S., Santos-Alvarez J., Martín-Romero C., Pérez-Pérez A., González-Yanes C., Sánchez-Margalet V. (2010). Role of leptin in the activation of immune cells. Mediat. Inflamm..

[41] Johnson R.B., Serio F.G. (2001). Leptin Within Healthy and Diseased Human Gingiva. J. Periodontol..

[42] Gundala R., Chava V.K., Ramalingam K. (2014). Association of Leptin in Periodontitis and Acute Myocardial Infarction. J. Periodontol..

[43] Tavares C.O., Rost F.L., Silva R.B., Dagnino A.P., Adami B., Schirmer H., de Figueiredo J.A., Souto A.A., Maito F.D., Campos M.M. (2019). Cross Talk between Apical Periodontitis and Metabolic Disorders: Experimental Evidence on the Role of Intestinal Adipokines and *Akkermansia muciniphila*. J. Endod..

[44] Gay I.C., Chen S., MacDougall M. (2007). Isolation and characterization of multipotent human periodontal ligament stem cells. Orthod. Craniofac. Res..

[45] Um S., Choi J.R., Lee J.H., Zhang Q., Seo B.M. (2011). Effect of leptin on differentiation of human dental stem cells. Oral Dis..

[46] Álvarez-Vásquez J.L., Bravo-Guapisaca M.I., Gavidia-Pazmiño J.F., Intriago-Morales R.V. (2021). Adipokines in dental pulp: Physiological, pathological, and potential therapeutic roles. J. Oral Biosci..

[47] El Karim I.A., Linden G.J., Irwin C.R., Lundy F.T. (2009). Neuropeptides Regulate Expression of Angiogenic Growth Factors in Human Dental Pulp Fibroblasts. J. Endod..

[48] Karaöz E., Doğan B.N., Aksoy A., Gacar G., Akyüz S., Ayhan S., Genç Z.S., Yürüker S., Duruksu G., Demircan P.Ç. (2010). Isolation and in vitro characterisation of dental pulp stem cells from natal teeth. Histochem. Cell Biol..

[49] Ide S., Tokuyama R., Davaadorj P., Shimozuma M., Kumasaka S., Tatehara S., Satomura K. (2011). Leptin and vascular endothelial growth factor regulate angiogenesis in tooth germs. Histochem. Cell Biol..

[50] Li W., Zhu W., Hou J., Huang B., Liu K., Meng H. (2014). Leptin and its receptor expression in dental and periodontal tissues of primates. Cell Tissue Res..

[51] Gronthos S., Brahim J., Li W., Fisher L.W., Cherman N., Boyde A., DenBesten P., Robey P.G., Shi S. (2002). Stem cell properties of human dental pulp stem cells. J. Dent. Res..

[52] Koyama N., Okubo Y., Nakao K., Bessho K. (2009). Evaluation of Pluripotency in Human Dental Pulp Cells. J. Oral Maxillofac. Surg..

[53] Martín-González J., Sánchez-Jiménez F., Pérez-Pérez A., Carmona-Fernández A., Sánchez-Margalet V., Segura-Egea J.J. (2013). Leptin expression in healthy and inflamed human dental pulp. Int. Endod. J..

[54] Martín-González J., Carmona-Fernández A., Pérez-Pérez A., Sánchez-Jiménez F., Sánchez-Margalet V., Segura-Egea J.J. (2015). Expression and immunohistochemical localization of leptin in human periapical granulomas. Med. Oral Patol. Oral Cir.

[55] Martín-González J., Pérez-Pérez A., Sánchez-Jiménez F., Carmona-Fernández A., Torres-Lagares D., Sánchez-Margalet V., Segura-Egea J.J. (2013). Leptin receptor is up-regulated in inflamed human dental pulp. J. Endod..

[56] Li W., Huang B., Liu K., Hou J., Meng H. (2015). Upregulated Leptin in Periodontitis Promotes Inflammatory Cytokine Expression in Periodontal Ligament Cells. J. Periodontol..

[57] Choi S.H., Jang J.H., Koh J.T., Chang H.S., Hwang Y.C., Hwang I.N., Lee B.N., Oh W.M. (2019). Effect of Leptin on Odontoblastic Differentiation and Angiogenesis: An In Vivo Study. J. Endod..

[58] Martín-González J., Pérez-Pérez A., Sánchez-Jiménez F., Díaz-Parrado E.M., de Miguel M., Sánchez-Margalet V., Segura-Egea J.J. (2015). Leptin promotes dentin sialophosphoprotein expression in human dental pulp. J. Endod..

[59] Martín-González J., Pérez-Pérez A., Cabanillas-Balsera D., Vilariño-García T., Sánchez-Margalet V., Segura-Egea J.J. (2019). Leptin stimulates DMP-1 and DSPP expression in human dental pulp via MAPK 1/3 and PI3K signaling pathways. Arch. Oral Biol..

[60] Ngo V.A., Jung J.Y., Koh J.T., Oh W.M., Hwang Y.C., Lee B.N. (2018). Leptin Induces Odontogenic Differentiation and Angiogenesis in Human Dental Pulp Cells via Activation of the Mitogen-activated Protein Kinase Signaling Pathway. J. Endod..

[61] Suzuki S., Haruyama N., Nishimura F., Kulkarni A.B. (2012). Dentin sialophosphoprotein and dentin matrix protein-1: Two highly phosphorylated proteins in mineralized tissues. Arch. Oral Biol..

[62] Deshpande A.S., Fang P.A., Zhang X., Jayaraman T., Sfeir C., Beniash E. (2011). Primary structure and phosphorylation of dentin matrix protein 1 (DMP1) and dentin phosphophoryn (DPP) uniquely determine their role in biomineralization. Biomacromolecules.

[63] Gronthos S., Mankani M., Brahim J., Robey P.G., Shi S. (2000). Postnatal human dental pulp stem cells (DPSCs) in vitro and in vivo. Proc. Natl. Acad. Sci. USA.

[64] Wei X., Ling J., Wu L., Liu L., Xiao Y. (2007). Expression of Mineralization Markers in Dental Pulp Cells. J. Endod..

[65] Martín-González J., Carmona-Fernández A., Pérez-Pérez A., Sánchez-Jiménez F., Sánchez-Margalet V., Segura-Egea J.J. (2015). Expression and immunohistochemical localization of leptin receptor in human periapical granuloma. Int. Endod. J..

[66] Da Rosa W.L.O., Piva E., Da Silva A.F. (2018). Disclosing the physiology of pulp tissue for vital pulp therapy. Int. Endod. J..

[67] Wei L., Chen Y., Zhang C., Liu M., Xiong H. (2019). Leptin induces IL-6 and IL-8 expression through leptin receptor Ob-Rb in human dental pulp fibroblasts. Acta Odontol. Scand..

[68] Grando Mattuella L., Westphalen Bento L., Poli de Figueiredo J.A., Eduardo Nör J., Borba de Araujo F., Christina Medeiros Fossati A. (2007). Vascular Endothelial Growth Factor and Its Relationship with the Dental Pulp. J. Endod..

[69] Murray P.E., Garcia-Godoy F., Hargreaves K.M. (2007). Regenerative Endodontics: A Review of Current Status and a Call for Action. J. Endod..

[70] Diogenes A., Henry M.A., Teixeira F.B., Hargreaves K.M. (2013). An update on clinical regenerative endodontics. Br. Dent. J..

[71] VanSaun M.N. (2013). Molecular pathways: Adiponectin and leptin signaling in cancer. Clin. Cancer Res..

[72] Kanoriya D., Pradeep A.R., Mallika A., Singhal S., Garg V. (2017). Correlation of crevicular fluid and serum levels of retinol-binding protein 4 and leptin in chronic periodontitis and obesity. Clin. Oral Investig..

[73] Kangarlou Haghighi A., Davar M., Kazem M., Dianat O. (2010). Presence of leptin in chronic periapical lesions. Iran. Endod. J..

[74] Nokhbehsaim M., Keser S., Nogueira A.V., Jäger A., Jepsen S., Cirelli J.A., Bourauel C., Eick S., Deschner J. (2014). Leptin Effects on the Regenerative Capacity of Human Periodontal Cells. Int. J. Endocrinol..

[75] Bilan P.J., Samokhvalov V., Koshkina A., Schertzer J.D., Samaan M.C., Klip A. (2009). Direct and macrophage-mediated actions of fatty acids causing insulin resistance in muscle cells. Arch. Physiol. Biochem..

[76] Hahn C.L., Best A.M., Tew J.G. (2000). Cytokine induction by *Streptococcus mutans* and pulpal pathogenesis. Infect. Immun..

[77] Liao F., Rabin R.L., Smith C.S., Sharma G., Nutman T.B., Farber J.M. (1999). CC-chemokine receptor 6 is expressed on diverse memory subsets of T cells and determines responsiveness to macrophage inflammatory protein 3 alpha. J. Immunol. Res..

[78] Dieu M.C., Vanbervliet B., Vicari A., Bridon J.M., Oldham E., Aït-Yahia S., Brière F., Zlotnik A., Lebecque S., Caux C. (1998). Selective recruitment of immature and mature dendritic cells by distinct chemokines expressed in different anatomic sites. J. Exp. Med..

[79] Farquharson A.J., Steele R.J., Carey F.A., Drew J.E. (2012). Novel multiplex method to assess insulin, leptin and adiponectinregulation of inflammatory cytokines associated with colon cancer. Mol. Biol. Rep..

[80] D’Aiuto F., Suvan J. (2012). Obesity, inflammation, and oral infections: Are microRNAs the missing link?. J. Dent. Res..

[81] Falagas M.E., Kompoti M. (2006). Obesity and infection. Lancet Infect. Dis..

[82] Pérez-Pérez A., Sánchez-Jiménez F., Vilariño-García T., Sánchez-Margalet V. (2020). Role of leptin in inflammation and vice versa. Int. J. Mol. Sci..

[83] Boesing F., Patiñeo J.S.R., Da Silva V.R.G., Moreira E.A.M. (2009). The interface between obesity and periodontitis with emphasis on oxidative stress and inflammatory response: Obesity Comorbidities. Obes. Rev..

[84] Cabanillas-Balsera D., Martín-González J., Montero-Miralles P., Sánchez-Domínguez B., Jiménez-Sánchez M.C., Segura-Egea J.J. (2019). Association between diabetes and nonretention of root filled teeth: A systematic review and meta-analysis. Int. Endod. J..

[85] Cabanillas-Balsera D., Segura-Egea J.J., Jiménez-Sánchez M.C., Areal-Quecuty V., Sánchez-Domínguez B., Montero-Miralles P., Saúco-Márquez J.J., Martín-González J. (2020). Cigarette Smoking and Root Filled Teeth Extraction: Systematic Review and Meta-Analysis. J. Clin. Med..

[86] Segura-Egea J.J., Martín-González J., Castellanos-Cosano L. (2015). Endodontic medicine: Connections between apical periodontitis and systemic diseases. Int. Endod. J..

[87] Segura-Egea J.J., Cabanillas-Balsera D., Jiménez-Sánchez M.C., Martín-González J. (2019). Endodontics and diabetes: Association versus causation. Int. Endod. J..

